# Sensitive detection of Aβ protofibrils by proximity ligation - relevance for Alzheimer's disease

**DOI:** 10.1186/1471-2202-11-124

**Published:** 2010-10-05

**Authors:** Masood Kamali-Moghaddam, Frida Ekholm Pettersson, Di Wu, Hillevi Englund, Spyros Darmanis, Anna Lord, Gholamreza Tavoosidana, Dag Sehlin, Sigrun Gustafsdottir, Lars NG Nilsson, Lars Lannfelt, Ulf Landegren

**Affiliations:** 1 Department of Genetics and Pathology, Molecular Medicine, Uppsala University, Uppsala, Sweden; 2 Department of Public Health and Caring Sciences, Molecular Geriatrics, Uppsala University, Uppsala, Sweden

## Abstract

**Background:**

Protein aggregation plays important roles in several neurodegenerative disorders. For instance, insoluble aggregates of phosphorylated tau and of Aβ peptides are cornerstones in the pathology of Alzheimer's disease. Soluble protein aggregates are therefore potential diagnostic and prognostic biomarkers for their cognate disorders. Detection of the aggregated species requires sensitive tools that efficiently discriminate them from monomers of the same proteins. Here we have established a proximity ligation assay (PLA) for specific and sensitive detection of Aβ protofibrils via simultaneous recognition of three identical determinants present in the aggregates. PLA is a versatile technology in which the requirement for multiple target recognitions is combined with the ability to translate signals from detected target molecules to amplifiable DNA strands, providing very high specificity and sensitivity.

**Results:**

For specific detection of Aβ protofibrils we have used a monoclonal antibody, mAb158, selective for Aβ protofibrils in a modified PLA, where the same monoclonal antibody was used for the three classes of affinity reagents required in the assay. These reagents were used for detection of soluble Aβ aggregates in solid-phase reactions, allowing detection of just 0.1 pg/ml Aβ protofibrils, and with a dynamic range greater than six orders of magnitude. Compared to a sandwich ELISA setup of the same antibody the PLA increases the sensitivity of the Aβ protofibril detection by up to 25-fold. The assay was used to measure soluble Aβ aggregates in brain homogenates from mice transgenic for a human allele predisposing to Aβ aggregation.

**Conclusions:**

The proximity ligation assay is a versatile analytical technology for proteins, which can provide highly sensitive and specific detection of Aβ aggregates - and by implication other protein aggregates of relevance in Alzheimer's disease and other neurodegenerative disorders.

## Background

In Alzheimer's disease (AD), brain deposits of extracellular amyloid-β (Aβ) and intracellular tau tangles are characteristic of the disease. Cerebrospinal fluid (CSF) is often investigated for levels of Aβ42, tau and phosho-tau in routine diagnostics of AD [1], where decreased Aβ42 and increased tau and/or phospho-tau (Thr_181P_) in CSF are indicative of the disease. These measures are reasonably good predictors of future conversion to AD among subjects with mild cognitive impairment, but they are not suitable to follow disease progression or to monitor drug intervention. Novel biomarkers are therefore needed, and evidence suggests that soluble, oligomeric aggregates of Aβ could be such a marker. For instance, levels of soluble forms of Aβ correlate more closely with disease severity than do the amounts of insoluble Aβ aggregates in the brain [2], and oligomeric Aβ has been shown to be neurotoxic, lead to synaptic dysfunction and to inhibit maintenance of hippocampal long-term potentiation [3-7]. Moreover, the so-called Arctic mutation causing early onset AD is located within the Aβ domain as are other mutations such as the Flemish, the Dutch and the Italian mutations, and this particular mutation has been shown to specifically enhance the formation of large soluble oligomers of Aβ (i. e. protofibrils), suggesting the notion that this Aβ species plays a central role in disease pathogenesis [8,9]. We previously developed a sensitive sandwich ELISA where the protofibril-selective mAb158 was used both as capture and detecting antibody [10]. Using this assay, the antibody used herein has been shown to detect Aβ protofibrils also in other, well-known, tg-mice such as PSAPP and tg2576 [11]. Here, we demonstrate that the proximity ligation assay (PLA) can provide even more sensitive detection of synthetic Aβ protofibrils.

PLA is an affinity-based technology enabling sensitive and specific detection of proteins in which the detection of proteins by sets of antibodies results in the formation of a specific DNA sequence by ligation of two parts. This sequence can then be amplified and quantified by methods such as real-time, PCR [12,13]. The technique makes use of affinity probes, typically antibodies coupled to oligonucleotides. Upon recognition of a common target molecule by a pair of such probes, the attached DNA strands are brought in proximity, allowing their free ends to be hybridized to a connector oligonucleotide that directs their joining by ligation. The reporter DNA strand that forms upon ligation can be amplified and quantified by methods such as real-time PCR. The assays can be performed in the homogenous phase with no need for washes or separations [12,13]. Alternatively, a solid support-bound affinity reagent can be used that offers the possibility to search for target molecules in larger sample volumes and to remove excess probes and undesired sample components before the ligation and amplification steps. This approach also adds specificity by requiring simultaneous recognition of three epitopes on the targets [14,15]. By using a single monoclonal antibody as the affinity reagent in all three affinity reagents required for solid-phase PLA (SP-PLA) we have achieved a highly sensitive assay that is specific for Aβ protofibrils, with excellent discrimination against monomers. We demonstrate that PLA is capable of detecting soluble Aβ aggregates in brains from mice transgenic for a pathogenic form of APP - the protein from which Aβ is derived.

## Results

**Figure 1:**
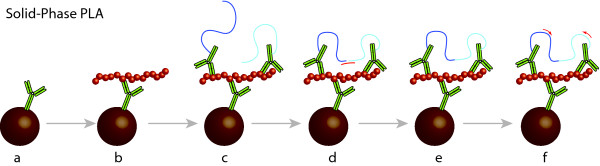
**Schematic illustration of SP-PLA**. (a) Capture antibodies are immobilized on a microparticulate solid support followed by (b) capture of target molecules from the biological sample. (c) Next, the beads are incubated with a pair of PLA probes - that is antibodies with attached oligonucleotides - where after excess probes and other reaction components are removed by washes. (d) Next a cocktail of reagents are added for probe ligation guided by a connector oligonucleotide, and for real-time PCR. (e) After a brief ligation step, (f) ligation products representing detected protofibrils are detected and quantified by real-time PCR.

We have developed a solid-phase form of PLA (Figure 1) using the mAb158 antibody for sensitive and specific detection of Aβ protofibrils, and we have compared this assay to the previously established sandwich ELISA using the same antibody.

**Figure 2:**
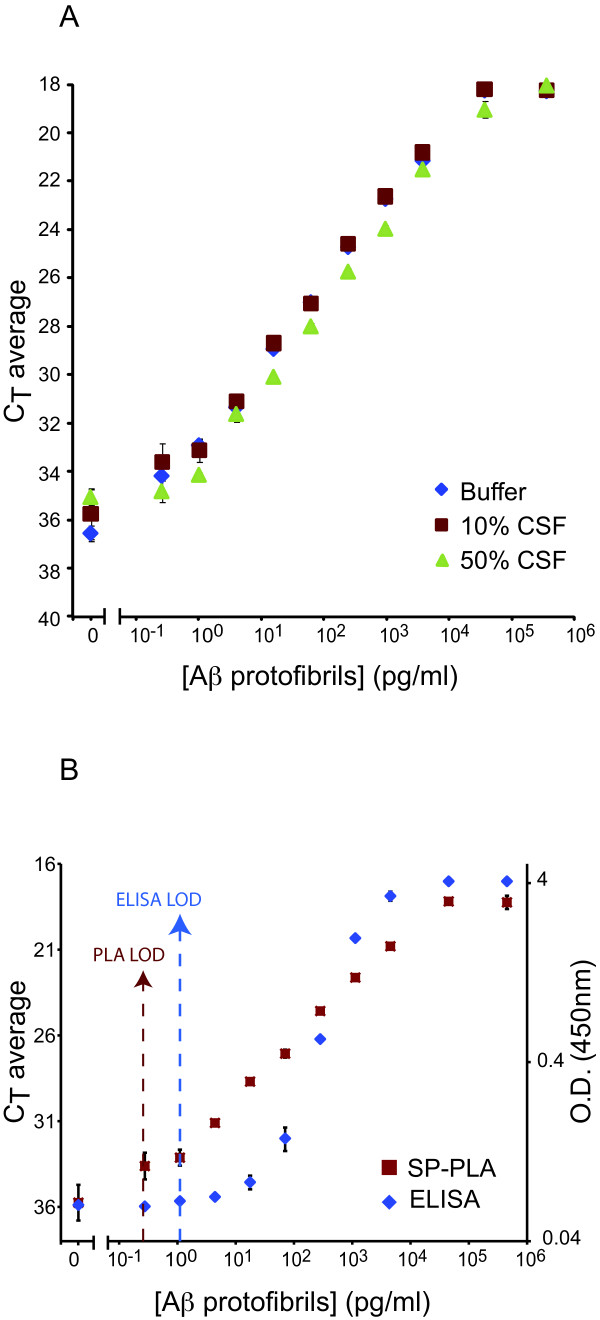
**Detection of Aβ protofibrils using SP-PLA**. (A) Assays were performed in buffer (blue diamonds), in 10% CSF (red squares), and in 50% CSF (green triangles). (B) Comparison of SP-PLA (red squares) versus ELISA (blue diamonds) for detection of Aβ protofibrils in 10% CSF. The X-axes show concentrations of Aβ protofibrils in pg/ml and the Y-axes show cycle threshold values for real-time PCR. In B the right Y-axis indicates the OD A_450_nm for ELISA. The vertical red and blue lines indicate the limit of detection for SP-PLA and ELISA, respectively, calculated as 2-fold standard deviations above the background signals. Error bars indicate standard deviations from the mean for triplicates for each reaction.

The SP-PLA has been previously shown to provide a broader dynamic range and a lower limit of detection for a wide range of proteins compared to standard sandwich ELISA protein assays [14]. Using the microparticle-based SP-PLA, Aβ was first captured by monoclonal antibodies immobilized on magnetic particles. Thereafter, the Aβ protofibrils were detected using a pair of PLA probes both utilizing the same monoclonal antibody as the capture reagent. This form of the assay thus ensures that only aggregates of Aβ are detected. Figure 2A illustrates the detection of Aβ protofibrils spiked in buffer, and in 10% and 50% CSF. The dynamic range of the assay on synthetic protofibrils extended over more than six orders of magnitude, and the limit of the detection was approximately 0.1 pg/ml for Aβ protofibrils spiked in buffer, and 0.27 pg/ml for Aβ protofibrils spiked in 10% and 50% CSF. These results represent a 4 to 10-fold greater sensitivity than the mAb158 ELISA. Figure 2B illustrates a comparison between SP-PLA and mAb158 ELISA for detection of synthetic Aβ protofibrils spiked in 10% CSF, where SP-PLA demonstrated a 4-fold lower limit of detection and a dynamic range four orders of magnitude greater than that of mAb158 ELISA.

**Figure 3:**
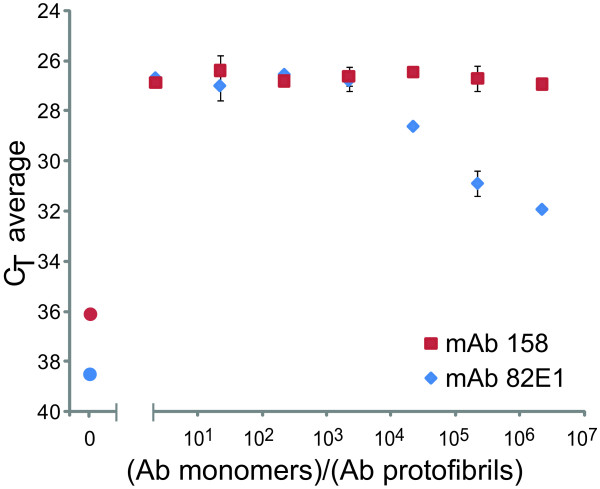
**SP-PLA-based detection of Aβ protofibrils in the presence of variable amounts of Aβ monomers**. The PLA signals for detection of 45 pg/ml Aβ protofibrils were not noticeably affected by the presence of an up to 2.2 million-fold molar excess of Aβ monomers for the protofibril-specific mAb158-PLA (red squares), and up to 22,000-fold molar excess of Aβ monomers for the 82E1-PLA (blue diamonds). The red and the blue circles indicate the assay background for mAb158 and 82E1, respectively. Error bars indicate standard deviations from the mean for triplicates for each reaction.

Since mAb158 also has affinity for monomeric Aβ- albeit with at least 200 times lower affinity than for protofibrils - it was of importance to examine whether the presence of Aβ monomers could interfere with the assay. We therefore analyzed Aβ protofibrils at a fixed concentration of 45 pg/ml (10 pM of original monomer concentration) in the presence of variable concentrations of monomeric Aβ1-16. Aβ1-16 was used in order to avoid trace amounts of Aβ aggregates of the monomers, and since both mAb158 and 82E1 bind this N-terminal region of Aβ [10]. As seen in Figure 3, detection was not compromised even at a 2.2 million-fold molar excess of Aβ monomers. When the SP-PLA was based on the monoclonal antibody, 82E1, which recognizes a linear, conformation independent Aβ epitope, a lower excess of 22,000-fold Aβ monomers was needed to compromise the signal.

**Figure 4:**
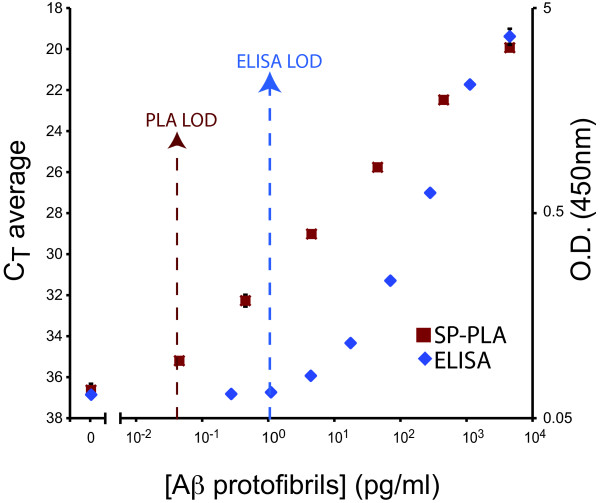
**SP-PLA was used to detect Aβ protofibrils in larger sample volumes**. Aβ protofibrils were spiked in 0.8 ml buffer and detected by SP-PLA. The result for SP-PLA is compared to standard mAb158 ELISA. The left Y-axis shows cycle threshold values for real-time PCR after SP-PLA, while the right Y-axis shows the OD A_450_nm for ELISA. The X-axis shows concentrations of Aβ protofibrils in pg/ml. Error bars indicate standard deviations from the mean for triplicates for each reaction.

One of the advantages of microparticle-based SP-PLA compared to standard ELISA and solution phase PLA lies in the possibility of using greater volumes of the samples. To take advantage of this opportunity we used a modified SP-PLA protocol in which 800 μl of a sample containing Aβ protofibrils was investigated, using a correspondingly higher amount of microparticles with immobilized capture antibodies. This should be compared to the 50 μl sample volume used in the conventional PLA protocol. In this form of the assay we achieved a further improved limit of detection of 0.04 pg/ml and a similar dynamic range as in the regular SP-PLA protocol. Compared to the mAb158 ELISA this assay thus detected 25-fold lower concentrations of synthetic Aβ protofibrils (Figure 4).

### Biologically derived Aβ protofibrils

**Figure 5:**
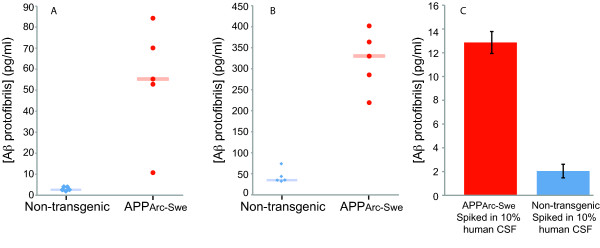
**Detection of endogenous Aβ protofibrils using SP-PLA**. Brain homogenates from five transgenic ArcSwe mice expressing elevated levels of protofibrils and five control mice were examined using SP-PLA (A) and the mAb158 ELISA (B). The blue and red rectangles indicate the median for control and transgenic-mice, respectively, revealing a significant difference between the two groups of mice. (C) Results of SP-PLA of brain homogenates from transgenic and non-transgenic mice, spiked in 10% human CSF. Error bars indicate standard deviations from the mean for triplicates for each reaction.

The Aβ protofibrils in the experiments reported so far were all prepared by *in vitro *aggregation of synthetic Aβ peptides. In order to investigate the ability of the SP-PLA to detect biologically derived soluble Aβ aggregates we used brain homogenates from five APP_Arc-Swe _mice [16], previously shown to have elevated levels of protofibrils [10,11]. In a blind-test, SP-PLA revealed concentrations of soluble Aβ aggregates in brain homogenates from the five transgenic mice, at levels ranging from 11 to 84 pg/ml. The samples from non-transgenic mice displayed background signals at 2 to 4 pg/ml (Figure 5A) and a similar difference was observed using the mAb158 ELISA. We note, however, that in both transgenic mice and controls the concentrations of protofibrils estimated by ELISA by reference to a standard dilution series of the *in vitro *aggregated form of the peptide were roughly 10-fold higher than those recorded by SP-PLA (Figure 5B). The differences in estimated concentrations in the two assays could be due to the requirement for antibody recognition of three identical determinants in SP-PLA while two determinants are bound in the sandwich ELISA, potentially rendering SP-PLA specific for larger aggregates. When we used a homogenous form of PLA where two recognition events are required to generate detection signals [12,13] the estimated concentrations of Aβ protofibrils - and possibly other lower molecular weight Aβ species, oligomers - in the tested samples were in the same range as those determined by ELISA (data not shown), supporting the notion that the SP-PLA form of the assays is limited to larger soluble Aβ aggregates. To establish the feasibility of demonstrating the presence of Aβ oligomers/protofibrils in human CSF we spiked brain homogenates either from a transgenic or a non-transgenic mouse in 10% human CSF. Figure 5C illustrates successful detection of endogenous Aβ aggregates from the transgenic mouse but not from the non-transgenic mouse after spiking the preparations in human CSF. The results illustrate the potential of the assay to detect low levels of Aβ oligomers in human bodily fluids.

## Discussion

Despite extensive efforts there remains an unmet need for highly specific and sensitive detection of soluble forms of the aggregated proteins that are found deposited in the brain in some neurodegenerative diseases. Sufficiently sensitive and specific methods could prove of great value in research, drug development, and for diagnostics and follow-up.

The proximity ligation assay has the advantage that it can be configured to require simultaneous binding to two or more epitopes in order to yield detectable signals. Herein we have used SP-PLA for specific detection of soluble Aβ aggregates via recognition by three reagents binding identical epitopes (Figure 1), thus, providing a stringent requirement for recognition of protein aggregates. This form of the assay involves one capture probe and two PLA probes, jointly ensuring low nonspecific background and exclusive detection of soluble aggregates where minimally three identical epitopes of the aggregated form of Aβ are recognized by the antibodies.

The other salient feature of PLA - the opportunity for DNA-based signal amplification using real-time PCR - serves to greatly enhance detection signals from the recognition reactions. The combination of highly specific recognition and amplified read-out allowed detection of protein protofibrils with increased sensitivity compared to our previously established ELISA (Figures 2B and 4). The SP-PLA allowed capture of Aβ protofibrils prior to extensive washes in a manner similar to a sandwich ELISA to remove excess detection reagents and components of the samples that might otherwise interfere with ligation, polymerization, or with fluorescence detection. We used five μl samples diluted to fifty μl in reaction buffer. SP-PLA served to further decrease background signals, providing for specific and near-linear detection of the protofibrils over a broad dynamic range.

The detection of different species of soluble Aβ aggregates in CSF could provide a means for unambiguous, potentially early diagnosis of AD. Other reported sensitive assays for detection of soluble aggregate proteins such as the bio-barcode assay rely on affinity binders specifically recognizing the oligomeric conformations of the target proteins [17], while in the SP-PLA-based approach the requirement for three recognition events also contribute to a high selectivity for protofibril conformations. A limited study using the SP-PLA tests for detection of Aβ aggregates in human CSF failed to reveal signals above background for either AD patients or controls, but this does not rule out protofibrils as a potential biomarker for the disease. It remains possible that further improved sensitivity will be required, or the lack of signal could be due to an inability of the antibody we used to detect the form of aggregated Aβ peptides in human CSF. Evidence has been presented that AD patients indeed do have aggregated forms of Aβ in CSF [18,19]. The detection of endogenous Aβ aggregates spiked in human CSF (Figure 5) indicates that SP-PLA performs adequately in this biological matrix.

## Conclusions

We have established that the PLA technique provides enhanced detection of aggregated Aβ proteins, offering high specificity, sensitivity and a wide dynamic range of detection. This makes PLA and further improvements thereof a promising tool for diagnostics in AD, and by extension also in other diseases characterized by increased levels of aggregated proteins.

## Methods

### Mouse brain homogenates and human CSF

Eight-month-old mice transgenic for human APP_Arc-Swe _(n = 5), and nontransgenic littermates (n = 5) [16] were anesthetized with 0.4 ml Avertin (25 mg/ml) and intracardially perfused with 0.9% saline solution. Frontal cortex from the brains was extracted as 1:10 (tissue weight/extraction volume ratio) in TBS (20 mmol/l Tris and 137 mmol/l NaCl, pH 7.6) with a complete protease inhibitor cocktail (Roche Diagnostics GmbH, Mannheim, Germany) using a tissue grinder with teflon pestle (2 × 10 strokes on ice). The homogenates were centrifuged at 100,000 g at 4°C for 60 min to obtain a preparation of TBS-soluble extracellular and cytosolic proteins. The supernatant was aliquoted and stored at -80°C prior to analysis.

CSF samples were collected by lumbar puncture at the Memory Clinic, Uppsala University Hospital, Uppsala, Sweden, as approved by the local ethics committee at Uppsala University (decision number 2005:244 and Ö 48-2005). Samples were centrifuged at 1,800 × *g *for 10 min to eliminate cells and insoluble material, and kept at -80°C until analysis. The CSF used in this study was pooled from 4 healthy individuals.

### Reagents

The monoclonal antibody mAb158, having selective affinity for Aβ in its protofibrillar conformation, has been described previously [10]. The monoclonal antibody 82E1, with affinity for a linear N-terminal Aβ neo-epitope, was purchased from IBL International (Hamburg, Germany). Synthetic Aβ42 was purchased from American Peptide (Sunnyvale, Ca, USA), and the Aβ protofibrils were prepared as previously described [20]. Briefly, lyophilized synthetic Aβ42wt was dissolved in 10 mM NaOH to a concentration of 100 μM, and then further diluted 1:1 with 2 × PBS (50 mM phosphate buffer and 100 mM NaCl, pH 7.4), and incubated at 37°C over night (ON) in the presence of 50 μM Docosahexaenoic acid (DHA) to stabilize the protofibrils. To remove fibrillar material the sample was centrifuged for 5 min at 17,900 × g before analyses. As determined by density gradient ultracentrifugation the mass of the Aβ protofibrils in this preparation are approximately 100-400 kDa (unpublished data). Lyophilized synthetic Aβ1-16wt peptide (Bachem, Bubendorf, Switzerland) was dissolved in 10 mM NaOH, prior to use, and diluted in 2 × PBS to a final concentration of 50 μM.

Oligonucleotide-streptavidin conjugates SLC1 (5'-streptavidin CGCATCGCCCTTGGACTACGACTGACGAACCGCTTTGCCTGACTGATCGCTAAATCGTG-3') and SLC2 (5'-TCGTGTCTAAAGTCCGTTACCTTGATTCCCCTAACCCTCTTGAAAAATTCGGCATCGGTGA-streptavidin 3') were purchased from Solulink (San Diego, CA, USA), and treated prior to use with free streptavidin to reduce the numbers of oligonucleotides per streptavidin tetramer, as described [14].

The same PCR forward primer, Biofwd, 5'-CATCGCCCTTGGACTACGA-3', PCR reverse primer, Biorev, 5'-GGGAATCAAGGTAACGGACTTTAG-3', and connector oligonucleotide, 5'-TACTTAGACACGACACGATTTAGTTT-3' were used in all PLA tests. These oligonucleotides were purchased from Biomers (Germany). A TaqMan probe (5' FAM-TGACGAACCGCTTTGCCTGA-MGB 3') was obtained from Applied Biosystems.

### Solid-phase proximity ligation assay

For all PLA reactions the oligonucleotide-streptavidin conjugates SLC1 and SLC2 were coupled to biotinylated antibodies by incubating identical volumes of 100 nM antibodies with 100 nM streptavidin-oligonucleotide conjugates for 1 h at room temperature. The antibody-oligonucleotide conjugates (PLA probes) thus obtained were used without purification after being separately diluted in PLA buffer (1 mM D-Biotin (Invitrogen), 0.1% purified BSA (New England Biolabs), 0.05% Tween 20 (Sigma-Aldrich), 100 nM goat serum IgG (Sigma-Aldrich), 0.1 μg/μl salmon sperm DNA (Invitrogen), 5 mM EDTA, 1 × PBS), and incubated for 15 min at room temperature prior to mixing the reagents to form a PLA probe mix.

Microparticle-based SP-PLA was carried out as described by Darmanis *et al. *[14], with some modifications as follows. Briefly, capture antibodies were bound to microparticles by using one mg of Dynabeads^® ^MyOne™ Streptavidin T1 microparticles (Invitrogen) that had been washed twice with 500 μl washing buffer (1 × PBS, 0.05% Tween 20 (Sigma-Aldrich)), using a 96-well plate magnet (Perkin Elmer) for separation of microparticles. The microparticles were mixed with 200 μl of 50 nM (1.5 μg) of the same biotinylated monoclonal antibody that was used for PLA probe, and incubated for 1 h at RT under rotation, followed by washes as above. The antibody-coated microparticles were suspended in 200 μl of storage buffer (1xPBS, 0.1% purified BSA (New England Biolabs)), and stored at 4°C for up to 2 months.

For each assay the storage buffer of one μl of antibody-coated microparticles (≈5 μg of microparticles and 7.5 ng of antibody) was replaced by 5 μl of PLA buffer, and the microparticles were mixed with 45 μl samples to be investigated for Aβ protofibrils. The binding reactions were incubated ON at 4°C or for 1.5 h at RT under rotation with similar efficiencies (data not shown). The microparticles were washed twice, and 50 μl of PLA probe mix at a concentration of 30 pM for each probe was added to each well, and incubated for 1.5 h at RT with rotation, followed by washing. Finally, 50 μl of ligation/PCR mix (1 × PCR buffer (Invitrogen), 2.5 mM MgCl_2 _(Invitrogen), 0.2 μM of each primer Biofwd and Biorev, 0.4 μM TaqMan probe, 0.08 mM ATP, 100 nM connector oligonucleotide, 0.2 mM dNTPs (containing dUTP) (Fermentas), 1.5 units Platinum *Taq *polymerase (Invitrogen), 0.5 units T4 DNA ligase (Fermentas), 0.1 units uracil-DNA glycosylase (Fermentas)) were added, followed by a 5 min incubation at room temperature for the proximity ligation step, before a real-time PCR was performed on an Mx-3000 real-time PCR instrument (Stratagene), with an initial incubation for 2 min at 95°C, and then 45 cycles of 15 s at 95°C and 1 min at 60°C.

For higher volume samples, 10 μl of antibody-coated microparticles were transferred to a 1.5 ml tube, and after removing the storage buffer the particles were mixed with 0.8 ml samples to be investigated for the presence of Aβ protofibrils, and incubated ON at 4°C with end-over-end rotation. The microparticles were collected by spinning at 15,000 rpm for 30 s, and washed twice. 50 μl PLA probe mix was added followed by incubation for 1.5 h at RT. Next, the microparticles were washed twice and transferred to optical PCR tubes, 50 μl ligation/PCR mix was added, and the real-time PCR was performed as described above.

### ELISA

The mAb158 sandwich ELISA was carried out as previously described [10]. In short, 96-well plates were coated with 200 ng/well of mAb158 at 4°C ON before being blocked with 1% BSA in PBS. 100 μl samples were added to the plate in triplicates and incubated for 2 h at RT. 0.5 μg/ml of biotinylated mAb158 was added and incubated for 1 h at RT, and then streptavidin-coupled horse radish peroxidase (Mabtech, Sweden) was added for 1 h at RT. K-blue enhanced (ANL produkter, Sweden) was used as a peroxidase substrate and the reactions were stopped with 1 M H_2_SO_4_. Wells were washed three times between each step after blocking the plates, and antibodies and samples were diluted in ELISA incubation buffer (PBS with 0.1% BSA, 0.05% Tween-20).

## Abbreviations

Aβ: Amyloid-β; AD: Alzheimer's disease; APP: Amyloid-β precursor protein; CSF: Cerebrospinal fluid; ELISA: Enzyme-linked immunosorbent assay; PBS: Phosphate buffered saline; PLA: Proximity ligation assay; SP-PLA: Solid-phase PLA

## Competing interests

U. Landegren and L. Lannfelt are founders and stockholders of Olink Bioscience and BioArctic Neuroscience, respectively.

## Authors' contributions

MKM designed the study, carried out PLA experiments, and wrote the manuscript; FEP participated in designing the study, carried out ELISA experiments and wrote parts of the manuscript; DW carried out PLA experiments and helped draft the manuscript; HE carried out initial PLA and ELISA experiments and helped draft the manuscript; SD participated in methods development of PLA and helped draft the manuscript; AL prepared and characterized mouse brain homogenates; GT contributed PLA reagents; DS carried out ELISA experiments, prepared and characterized mouse brain homogenates; SG contributed PLA regents and methods development; LNGN provided samples from mice and helped draft the manuscript; LL provided antibodies and human CSF samples and helped draft the manuscript; and UL participated in designing the study and writing the manuscript. All authors read and approved the final manuscript.
